# Supplementary material for: Inter-continental variability in the relationship of oxidative potential and cytotoxicity with PM2.5 mass
Source: Nat Commun. 2024 Jun 19;15:5263. doi: 10.1038/s41467-024-49649-4 (PMC11187120; doi:10.1038/s41467-024-49649-4)
Supplement: Supplementary file 1 — Supplementary Information [file 41467_2024_49649_MOESM1_ESM.pdf]

Supplementary Information

Inter-continental variability in the relationship of oxidative potential and cytotoxicity with $PM_{2.5}$ mass

Sudheer Salana[1], Haoran Yu[2,1], Zhuying Dai[1], P.S. Ganesh Subramanian[1], Joseph V. Puthussery[3,1], Yixiang Wang[4,1], Ajit Singh[5,6], Francis D. Pope[5], Manuel A. Leiva G.[7], Neeraj Rastogi[8], Sachchida Nand Tripathi[9,10], Rodney J. Weber[11], Vishal Verma[1]*

[1]Department of Civil and Environmental Engineering, University of Illinois at Urbana Champaign, Urbana, 61801, United States.

[2]Department of Civil and Environmental Engineering, University of Alberta, Edmonton, AB, Canada.

[3]Center for Aerosol Science and Engineering, Department of Energy, Environmental and Chemical Engineering, Washington University in St. Louis, St. Louis, MO, 63130, USA

[4]College of Health, Lehigh University, Bethlehem, PA, 18015, USA

[5]School of Geography, Earth and Environmental Sciences, University of Birmingham, Birmingham B15 2TT, UK

[6] Institute of Applied Health Research, University of Birmingham, Edgbaston, Birmingham B15 2TT, UK

[7]Department of Chemistry, Faculty of Science, Universidad de Chile, Las Palmeras 3425, Ñuñoa, Santiago, RM, Chile

[8]Geosciences Division, Physical Research Laboratory, Ahmedabad, 380009, India.

[9]Department of Civil Engineering, Indian Institute of Technology Kanpur, Kanpur 208016, India.

[10]Department of Sustainable Energy Engineering, Indian Institute of Technology Kanpur, Kanpur 208016, India.

[11]School of Earth and Atmospheric Sciences, Georgia Institute of Technology, Atlanta, GA 30332, USA

Pages = 28

Number of Tables = 5

Number of Figures = 10

*Corresponding Author
Vishal Verma
Associate Professor,
Department of Civil and Environmental Engineering,
University of Illinois at Urbana-Champaign
205 N. Mathews Ave
Urbana, IL 61801
email: vverma@illinois.edu
phone: (217) 265-6703

Supplementary Table 1: Dates of sample collection (yyyy-dd-mm), sample duration, and average $PM_{2.5}$ mass concentrations during sampling at all the sites (Blank spaces indicate that no collection of the sample occurred on that date (due to several reasons such as unfavorable weather conditions, broken sampler, etc.)

Region	Site	Season	Sampling period starting date	Sampling duration	Average $PM_{2.5}$ mass concentration during sampling
			(yyyy-dd-mm)	(h)	($\mu g/m^3$)
Midwest US	Chicago (CHI)	Summer 2018	2018-22-05	72	8.4
			2018-29-05	72	11.0
			2018-05-06	72	10.0
			2018-12-06	72	12.2
			2018-19-06	72	10.3
			2018-26-06	72	12.0
			2018-03-07	72	10.8
			2018-10-07	72	11.5
			2018-17-07	72	7.6
			2018-24-07	72	
			2018-31-07	72	13.2
			2018-07-08	72	10.0
			2018-14-08	72	19.0
			2018-21-08	72	14.4
			2018-28-08	72	5.7
		Fall 2018	2018-04-09	72	7.1
			2018-11-09	72	9.5
			2018-18-09	72	10.6
			2018-25-09	72	
			2018-02-10	72	
			2018-09-10	72	6.5
			2018-16-10	72	12.4
			2018-23-10	72	8.7
			2018-30-10	72	11.4
			2018-06-11	72	7.6
			2018-13-11	72	16.6
			2018-20-11	72	13.1
			2018-27-11	72	15.9
		Winter 2019	2018-04-12	72	11.0
			2018-11-12	72	
			2018-18-12	72	
			2018-25-12	72	
			2019-01-01	72	
			2019-08-01	72	
			2019-15-01	72	
			2019-22-01	72	18.2
			2019-29-01	72	17.0
			2019-05-02	72	9.5
			2019-12-02	72	12.5
			2019-19-02	72	18.4
			2019-26-02	72	15.4
		Spring 2019	2019-05-03	72	18.1
			2019-12-03	72	
			2019-19-03	72	19.1
			2019-26-03	72	14.0
			2019-02-04	72	14.5
			2019-09-04	72	8.7
			2019-16-04	72	12.0
			2019-23-04	72	11.7
			2019-30-04	72	6.9
			2019-07-05	72	7.0
			2019-14-05	72	17.4
			2019-21-05	72	12.1
			2019-28-05	72	9.8

Supplementary Table 1 (Contd.):

Region	Site	Season	Sampling period starting date	Sampling duration	Average $PM_{2.5}$ mass concentration during sampling
			(yyyy-dd-mm)	(h)	($\mu g/m^3$)
Midwest US	St Louis (STL)	Summer 2018	2018-22-05	72	18.0
			2018-29-05	72	13.6
			2018-05-06	72	20.4
			2018-12-06	72	15.7
			2018-19-06	72	11.9
			2018-26-06	72	10.4
			2018-03-07	72	20.1
			2018-10-07	72	17.0
			2018-17-07	72	15.8
			2018-24-07	72	15.9
			2018-31-07	72	14.4
			2018-07-08	72	13.7
			2018-14-08	72	10.1
			2018-21-08	72	15.1
			2018-28-08	72	9.2
		Fall 2018	2018-04-09	72	11.7
			2018-11-09	72	17.1
			2018-18-09	72	15.7
			2018-25-09	72	8.9
			2018-02-10	72	11.0
			2018-09-10	72	
			2018-16-10	72	10.0
			2018-23-10	72	13.2
			2018-30-10	72	8.0
			2018-06-11	72	
			2018-13-11	72	
			2018-20-11	72	16.9
			2018-27-11	72	18.3
		Winter 2019	2018-04-12	72	9.2
			2018-11-12	72	13.3
			2018-18-12	72	15.0
			2018-25-12	72	10.8
			2019-01-01	72	9.9
			2019-08-01	72	7.8
			2019-15-01	72	12.3
			2019-22-01	72	12.8
			2019-29-01	72	11.5
			2019-05-02	72	9.5
			2019-12-02	72	9.6
			2019-19-02	72	13.4
			2019-26-02	72	18.1
		Spring 2019	2019-05-03	72	17.5
			2019-12-03	72	10.0
			2019-19-03	72	12.7
			2019-26-03	72	15.4
			2019-02-04	72	15.1
			2019-09-04	72	21.7
			2019-16-04	72	11.5
			2019-23-04	72	9.2
			2019-30-04	72	
			2019-07-05	72	11.0
			2019-14-05	72	
			2019-21-05	72	
			2019-28-05	72	

Supplementary Table 1 (Contd.):

Region	Site	Season	Sampling period starting date	Sampling duration	Average $PM_{2.5}$ mass concentration during sampling
			(yyyy-dd-mm)	(h)	(μg/m^3)
Midwest US	Indianapolis (IND)	Summer 2018	2018-22-05	72	11.1
			2018-29-05	72	11.5
			2018-05-06	72	11.9
			2018-12-06	72	11.6
			2018-19-06	72	8.8
			2018-26-06	72	6.8
			2018-03-07	72	19.5
			2018-10-07	72	15.2
			2018-17-07	72	10.6
			2018-24-07	72	13.8
			2018-31-07	72	11.6
			2018-07-08	72	6.7
			2018-14-08	72	17.0
			2018-21-08	72	12.4
			2018-28-08	72	9.7
		Fall 2018	2018-04-09	72	14.2
			2018-11-09	72	11.3
			2018-18-09	72	17.1
			2018-25-09	72	8.5
			2018-02-10	72	8.7
			2018-09-10	72	5.7
			2018-16-10	72	10.4
			2018-23-10	72	10.2
			2018-30-10	72	9.3
			2018-06-11	72	6.3
			2018-13-11	72	15.4
			2018-20-11	72	20.1
			2018-27-11	72	11.8
		Winter 2019	2018-04-12	72	9.2
			2018-11-12	72	15.1
			2018-18-12	72	12.8
			2018-25-12	72	11.2
			2019-01-01	72	7.7
			2019-08-01	72	8.5
			2019-15-01	72	11.6
			2019-22-01	72	10.9
			2019-29-01	72	10.0
			2019-05-02	72	7.3
			2019-12-02	72	9.9
			2019-19-02	72	12.9
			2019-26-02	72	16.3
		Spring 2019	2019-05-03	72	13.3
			2019-12-03	72	10.2
			2019-19-03	72	17.2
			2019-26-03	72	13.2
			2019-02-04	72	12.1
			2019-09-04	72	12.5
			2019-16-04	72	12.2
			2019-23-04	72	10.0
			2019-30-04	72	9.9
			2019-07-05	72	11.9
			2019-14-05	72	14.1
			2019-21-05	72	9.3
			2019-28-05	72	12.8

Supplementary Table 1 (Contd.):

Region	Site	Season	Sampling period starting date	Sampling duration	Average $PM_{2.5}$ mass concentration during sampling
			(yyyy-dd-mm)	(h)	(µg/m^3)
Midwest US	Champaign (CMP)	Summer 2018	2018-22-05	72	15.4
			2018-29-05	72	10.3
			2018-05-06	72	12.9
			2018-12-06	72	6.9
			2018-19-06	72	10.1
			2018-26-06	72	5.5
			2018-03-07	72	8.1
			2018-10-07	72	13.2
			2018-17-07	72	8.6
			2018-24-07	72	12.8
			2018-31-07	72	11.6
			2018-07-08	72	13.0
			2018-14-08	72	20.2
			2018-21-08	72	15.4
			2018-28-08	72	7.2
		Fall 2018	2018-04-09	72	5.9
			2018-11-09	72	2.0
			2018-18-09	72	2.5
			2018-25-09	72	6.2
			2018-02-10	72	9.1
			2018-09-10	72	5.1
			2018-16-10	72	6.3
			2018-23-10	72	9.3
			2018-30-10	72	
			2018-06-11	72	7.3
			2018-13-11	72	17.7
			2018-20-11	72	
			2018-27-11	72	10.7
		Winter 2019	2018-04-12	72	8.4
			2018-11-12	72	14.3
			2018-18-12	72	8.3
			2018-25-12	72	7.4
			2019-01-01	72	9.1
			2019-08-01	72	6.4
			2019-15-01	72	10.9
			2019-22-01	72	11.1
			2019-29-01	72	7.1
			2019-05-02	72	8.2
			2019-12-02	72	9.2
			2019-19-02	72	12.2
			2019-26-02	72	16.7
		Spring 2019	2019-05-03	72	13.8
			2019-12-03	72	10.0
			2019-19-03	72	11.9
			2019-26-03	72	14.6
			2019-02-04	72	16.9
			2019-09-04	72	9.2
			2019-16-04	72	
			2019-23-04	72	14.3
			2019-30-04	72	7.9
			2019-07-05	72	14.2
			2019-14-05	72	9.9
			2019-21-05	72	8.3
			2019-28-05	72	7.9

Supplementary Table 1 (Contd.):

Region	Site	Season	Sampling period starting date	Sampling duration	Average $PM_{2.5}$ mass concentration during sampling
			(yyyy-dd-mm)	(h)	(µg/m^3)
Midwest US	Bondville (BND)	Summer 2018	2018-22-05	72	
			2018-29-05	72	
			2018-05-06	72	8.9
			2018-12-06	72	9.4
			2018-19-06	72	
			2018-26-06	72	11.3
			2018-03-07	72	8.3
			2018-10-07	72	
			2018-17-07	72	
			2018-24-07	72	10.8
			2018-31-07	72	10.9
			2018-07-08	72	9.3
			2018-14-08	72	14.1
			2018-21-08	72	12.8
			2018-28-08	72	7.9
		Fall 2018	2018-04-09	72	8.5
			2018-11-09	72	8.0
			2018-18-09	72	14.1
			2018-25-09	72	
			2018-02-10	72	
			2018-09-10	72	6.8
			2018-16-10	72	5.9
			2018-23-10	72	8.7
			2018-30-10	72	9.2
			2018-06-11	72	5.2
			2018-13-11	72	13.4
			2018-20-11	72	16.1
			2018-27-11	72	10.8
		Winter 2019	2018-04-12	72	7.2
			2018-11-12	72	13.3
			2018-18-12	72	12.3
			2018-25-12	72	6.2
			2019-01-01	72	6.9
			2019-08-01	72	5.0
			2019-15-01	72	
			2019-22-01	72	
			2019-29-01	72	5.8
			2019-05-02	72	8.1
			2019-12-02	72	9.4
			2019-19-02	72	6.8
			2019-26-02	72	13.1
		Spring 2019	2019-05-03	72	10.1
			2019-12-03	72	7.2
			2019-19-03	72	12.0
			2019-26-03	72	8.7
			2019-02-04	72	11.6
			2019-09-04	72	6.6
			2019-16-04	72	7.3
			2019-23-04	72	11.3
			2019-30-04	72	7.2
			2019-07-05	72	7.3
			2019-14-05	72	9.7
			2019-21-05	72	7.1
			2019-28-05	72	13.4

Supplementary Table 1 (Contd.):

Region	Site	Season	Sampling period starting date	Sampling duration (h)	Average $PM_{2.5}$ mass concentrations during sampling ($\mu g/m^3$)
Southeast US	Altanta	Winter	2018-26-01	24	6
			2018-16-03	24	13
			2018-07-11	24	8
			2018-13-11	24	20
			2018-17-11	24	22
			2018-18-11	24	10
			2018-19-11	24	11
			2018-29-11	24	16
			2018-02-12	24	8
			2018-25-12	24	11.5
		Summer	2018-07-06	24	17
			2018-08-06	24	18
			2018-09-06	24	23
			2018-10-06	24	30
			2018-20-06	24	16
			2018-12-07	24	22
			2018-15-08	24	20
			2018-23-08	24	21
			2018-26-08	24	19
			2018-28-08	24	17
Birmingham (West Midlands, UK)	EROS	Fall	2019-12-08	120	6.4
			2019-17-08	120	7.1
			2019-22-08	120	16.9
			2019-27-08	120	5.9
			2019-01-09	120	5.6
			2019-06-09	120	5.1
			2019-11-09	120	5.3
			2019-16-09	120	9.1
			2019-21-09	120	5.4
			2019-26-09	120	2.4
			2019-01-10	120	5.5
			2019-06-09	120	3.6
			2019-11-09	120	3.0
	BROS		2019-16-09	120	5.8
			2019-21-09	120	8.3
			2019-26-09	120	9.3
			2019-01-11	120	7.5
			2019-06-11	120	6.4
			2019-11-11	120	6.0
			2019-16-11	120	6.7
			2019-21-11	120	7.1
India	Patiala	Winter	2011-26-10	10	344
			2011-27-10	10	404
			2011-20-12	10	258
			2012-02-01	10	304
			2012-04-02	10	229
	Ahmedabad		2020-15-11	24	221
			2020-17-11	24	248
			2020-23-11	24	194
	Faridabad		2019-03-11	24	275.2
			2019-25-11	24	80.17
			2019-12-11	24	161.7
			2019-29-10	24	226.66
			2019-06-11	24	111.2
	Hisar		2019-25-11	24	128.8
			2019-31-10	24	201.93
			2019-12-11	24	560.58
			2019-27-10	24	234.49
			2019-06-11	24	95.69

Supplementary Table 1 (Contd.):

Region	Site	Season	Sampling period starting date	Sampling duration (h)	Average $PM_{2.5}$ mass concentrations during sampling (μg/m^3)
Chile	Santiago	Summer	2020-03-01	24	16.4
			2020-07-01	24	13.5
			2020-10-01	24	12.1
			2020-13-01	24	14.2
			2019-31-12	24	7.7
			2019-22-12	24	15.2
			2019-19-12	24	10.5
			2019-16-12	24	12.1
			2019-10-12	24	14.1
			2019-07-12	24	11.4
		Spring	2019-04-10	24	15.8
			2019-07-10	24	14.7
			2019-10-10	24	20.3
			2019-28-09	24	22.8
			2019-25-09	24	30.0
			2019-22-09	24	17.9
			2019-19-09	24	14.6
			2019-16-09	24	12.8
			2019-13-09	24	28.1
			2019-10-09	24	16.9
		Winter	2019-31-08	24	28.7
			2019-29-08	24	13.5
			2019-25-08	24	30.3
			2019-23-08	24	33.4
			2019-17-08	24	33.9
			2019-08-08	24	30.7
			2019-05-08	24	39.2
			2019-30-07	24	42.9
			2019-21-07	24	17.6
			2019-18-07	24	67.3
			2019-15-07	24	64.8
			2019-12-07	24	71.1
			2019-09-07	24	63.8
			2019-06-07	24	45.2
			2019-03-07	24	56.3
			2019-30-06	24	31.0
			2019-27-06	24	71.7
			2019-24-06	24	31.6
			2019-16-06	24	17.0
			2019-11-06	24	27.0
		Fall	2019-19-05	24	32.4
			2019-16-05	24	59.0
			2019-13-05	24	35.9
			2019-10-05	24	37.7
			2019-07-05	24	47.1
			2019-04-05	24	24.5
			2019-01-05	24	29.3
			2019-17-04	24	30.9
			2019-14-04	24	65.0
			2019-11-04	24	36.8

Supplementary Table 1 (Contd.):

Region	Site	Season	Sampling period starting date	Sampling duration (h)	Average $PM_{2.5}$ mass concentrations during sampling (µg/m^3)
Chile	Chillan	Fall	2019-04-05	24	15.0
			2019-07-05	24	29.6
			2019-11-05	24	82.9
			2019-13-05	24	37.5
			2019-15-05	24	107.3
			2019-18-05	24	20.7
			2019-21-05	24	36.0
			2019-24-05	24	127.1
			2019-26-05	24	21.2
			2019-29-05	24	17.0
		Winter	2019-03-07	24	79.2
			2019-07-07	24	32.4
			2019-09-07	24	23.7
			2019-12-07	24	32.0
			2019-15-07	24	26.5
			2019-19-07	24	64.3
			2019-21-07	24	11.0
			2019-25-07	24	39.6
			2019-27-07	24	48.5
			2019-30-07	24	14.4
		Spring	2019-20-09	24	8.3
			2019-22-09	24	9.7
			2019-25-09	24	12.4
			2019-29-09	24	10.8
			2019-05-10	24	17.7
			2019-07-10	24	6.1
			2019-10-10	24	19.4
			2019-13-10	24	11.4
			2019-16-10	24	11.2
			2019-18-09	24	12.2
		Summer	2018-01-12	24	8.3
			2018-03-12	24	10.9
			2018-07-12	24	12.6
			2018-09-12	24	5.5
			2018-12-12	24	6.1

Supplementary Method 1: Preparation of DCFH-DA working solution.

The working solution of DCFH-DA was prepared from a 45 mM stock solution of DCFH-DA which was prepared in N, N-Dimethylformamide (DMF). The stock solution was aliquoted into different vials (30 µL per vial), stored in a freezer (at −20 ºC), and used within a month. To prepare the final probe solution, a portion of the content of one vial (i.e., 25 µL of 45 mM DCFH-DA) was diluted 100 times (final concentration of DCFH-DA working solution = 450 µM) just before the experiment, using a 10X concentration of Salt glucose medium (SGM). The SGM was prepared according to the composition discussed in Klein et al. (2002)[1], and was provided by the Cell Media Facility at UIUC.

Supplementary Method 2: **Stability of the working solution of DCFH-DA**

To determine the stability of DCFH-DA for cellular OP measurements, the working solution of DCFH-DA (450 µM) was prepared, and its absolute fluorescence was measured at various time points: 0, 1, 3, 4, 5, 6, 8, 12, 25, 48 and 72 h. Concurrently, DCFH was prepared by deacetylating DCFH-DA (450 µM) using the method described in Reiniers et al.[2] and mixed with 100 µM H_2O_2 (working as a surrogate for the ROS generated by the cells). The fluorescence of this mixture was also measured at the same time points (i.e., 0, 1, 3, 4, 5, 6, 8, 12, 25, 48, and 72 h). As shown in Supplementary Fig.1, the absolute fluorescence of DCFH-DA was relatively negligible until 25 h and started to gradually increase thereafter. However, the fluorescence of the DCFH and H_2O_2 mixture attained its maximum fluorescence within 8 h and stayed constant thereafter, indicating that the entire DCFH had reacted with H_2O_2 to form DCF. Therefore, it can be concluded that the fluorescence of DCF formed due to the reaction between DCFH and ROS remains stable till at least 24 h. However, the working solution of DCFH-DA could become unstable and undergo autoxidation for an incubation period of more than 24 h.

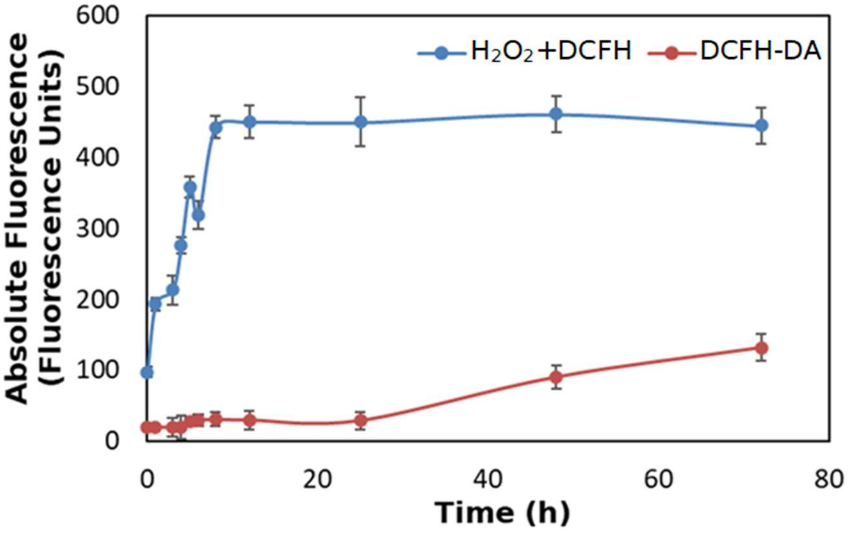

Supplementary Fig. 1: Variation in the absolute fluorescence of DCFH-DA over time. The blue line represents the fluorescence of DCFH treated with H_2O_2, whereas the red line represents the fluorescence of the working solution of DCFH-DA without any treatment.

Supplementary Table 2: Uncertainty (%) estimates for various measurements, i.e., $PM_{2.5}$ mass, chemical components, OP and cytotoxicity*

Region	$PM_{2.5}$ metric		Instrument used for the measurement	Total Uncertainty in the measurement (%)
**Midwest US**	$PM_{2.5}$ mass		Tisch Environmental Hi Vol Sampler for filter collection and Satorius, A120S for filter weighing	7.8
	OP or Toxicity endpoints	Cytotoxicity	SpectraMax microplate reader (Molecular Devices, CA)	9.9
		$OP^{OH}v$	SAMERA	10.2
		$OP^{GSH}v$	SAMERA	11.8
		$OP^{C}v$	Bench-top spectrofluorometer (RF-5301 pc, Shimadzu Co., Japan)	6.9
		$OP^{DTT}v$	SAMERA	8.9
	Chemical Composition	Metals	NexION 300X ICP-MS; Perkin Elmer, Waltham, MA	10.0
		WSOC	TOC Analyzer , Shimadzu Co., Japan	9.5
**West Midlands**	$PM_{2.5}$ mass		DIGITEL Hi Vol Sampler for filter collection and Sartorius, LE324S for filter weighing	9.9
	OP or Toxicity endpoints	Cytotoxicity	SpectraMax microplate reader (Molecular Devices, CA)	9.3
		$OP^{OH}v$	SAMERA	11.3
		$OP^{GSH}v$	SAMERA	12.8
		$OP^{C}v$	Bench-top spectrofluorometer (RF-5301 pc, Shimadzu Co., Japan)	8.4
		$OP^{DTT}v$	SAMERA	10.2
	Chemical Composition	Metals	NexION 300X ICP-MS; Perkin Elmer, Waltham, MA	11.7
		WSOC	TOC Analyzer , Shimadzu Co., Japan	11.3
**Chile**	$PM_{2.5}$ mass		MCV, S.A. Hi Vol Sampler model CAV-A /Mb for filter collection and Radwag XA 110/4Y for filter weighing	7.2
	OP or Toxicity endpoints	Cytotoxicity	SpectraMax microplate reader (Molecular Devices, CA)	7.3
		$OP^{OH}v$	SAMERA	9.8
		$OP^{GSH}v$	SAMERA	11.5
		$OP^{C}v$	Bench-top spectrofluorometer (RF-5301 pc, Shimadzu Co., Japan)	6.2
		$OP^{DTT}v$	SAMERA	8.4
	Chemical Composition	Metals	NexION 300X ICP-MS; Perkin Elmer, Waltham, MA	9.6
		WSOC	TOC Analyzer , Shimadzu Co., Japan	9.0
**Atlanta**	$PM_{2.5}$ mass		Thermo Anderson Hi Vol Sampler for filter collection and Tapered element oscillating microbalance (TEOM) , Thermo Scientific TEOM 1400a for PM2.5 mass measurement	10.0
	OP or Toxicity endpoints	Cytotoxicity	SpectraMax microplate reader (Molecular Devices, CA)	6.5
		$OP^{OH}v$	SAMERA	9.1
		$OP^{GSH}v$	SAMERA	10.9
		$OP^{C}v$	Bench-top spectrofluorometer (RF-5301 pc, Shimadzu Co., Japan)	5.1
		$OP^{DTT}v$	SAMERA	7.6
	Chemical Composition	Metals	NexION 300X ICP-MS; Perkin Elmer, Waltham, MA	11.8
		WSOC	TOC Analyzer , Shimadzu Co., Japan	11.4
**India**	$PM_{2.5}$ mass		Thermo Scientific Hi Vol Sampler for filter collection and Sartorius, LA130S-F for filter weighing	10.3
	OP or Toxicity endpoints	Cytotoxicity	SpectraMax microplate reader (Molecular Devices, CA)	6.7
		$OP^{OH}v$	SAMERA	9.3
		$OP^{GSH}v$	SAMERA	11.0
		$OP^{C}v$	Bench-top spectrofluorometer (RF-5301 pc, Shimadzu Co., Japan)	5.3
		$OP^{DTT}v$	SAMERA	7.8
	Chemical Composition	Metals	NexION 300X ICP-MS; Perkin Elmer, Waltham, MA	12.1
		WSOC	TOC Analyzer , Shimadzu Co., Japan	11.6

*Uncertainty in $PM_{2.5}$ mass measurements (σ_m) was calculated as follows:

$$\sigma_m = \sqrt{(\sigma_f)^2 + (\sigma_w)^2}$$

where, σ_f is the uncertainty in the volume of air sampled (2-7%; depending on the sampler) and σ_w is the uncertainty in weighing filters (6-10%; depending on the weighing balance). At Atlanta site, uncertainty in $PM_{2.5}$ mass measurements was the uncertainty of Tapered Element Oscillating Microbalance (TEOM) as provided by the manufacturer.

Uncertainties in OPv and CDv measurements (σ_{OP} and σ_{CD}) were calculated as follows:

$$\sigma_{OP} (or\ \sigma_{CD}) = \sqrt{(\sigma_f)^2 + (\sigma_{me})^2 + (\sigma_e)^2}$$

where, σ_{me} is the uncertainty of the instrument used for the specific measurement and σ_e is the uncertainty in $PM_{2.5}$ extraction (2%). The uncertainties (σ_{me}) for SAMERA, used for acellular OP [OP^{OH}v (9%), OP^{GSH}v (11%), and OP^{DTT}v (7%)] measurements were calculated from the accuracy and precision data reported in our previous publication[3]. The uncertainties for the instruments used for measuring OP^{C}v [Benchtop spectrofluorometer (4%)] and CDv [SpectraMax microplate reader (6%)] were calculated from the accuracy and precision of the instruments provided by the manufacturer.

Uncertainty in the concentrations of chemical species (σ_c) was calculated as follows:

$$\sigma_c = \sqrt{(\sigma_m)^2 + (\sigma_e)^2 + (\sigma_i)^2}$$

where, σ_i is the uncertainty of ICP-MS (6%), or TOC analyzer (5%) used for metals and WSOC measurements, respectively, as calculated from the accuracy and precision data of the instruments provided by the manufacturer.

Supplementary Table 3: *p* values for the site-wise and within-site comparison of CoVs for different $PM_{2.5}$ endpoints. *p* values ≤ 0.05 are shown in bold.

	Endpoint	Site	Midwest US	West Midlands	Chile	Atlanta	India
***p* values for comparison of CoVs for different $PM_{2.5}$ endpoints across different regions**	Mass	Midwest US		**0.04**	**<0.001**	0.44	**0.01**
		West Midlands			0.07	0.48	0.74
		Chile				**0.02**	0.15
		Atlanta					0.31
	CDv	Midwest US		**0.02**	**0.01**	0.52	0.98
		West Midlands			0.67	0.10	0.25
		Chile				0.16	0.35
		Atlanta					0.63
	$OP^{OH}v$	Midwest US		0.64	**0.03**	**0.04**	0.41
		West Midlands			0.24	**0.05**	0.71
		Chile				**0.02**	0.18
		Atlanta					0.11
	$OP^{GSH}v$	Midwest US		0.45	0.07	0.41	0.34
		West Midlands			0.83	0.92	0.71
		Chile				0.95	0.79
		Atlanta					0.79
	$OP^{C}v$	Midwest US		0.28	0.71	0.16	0.84
		West Midlands			0.46	0.06	0.43
		Chile				0.13	0.72
		Atlanta					0.26
	$OP^{DTT}v$	Midwest US		0.57	**<0.001**	0.28	**0.04**
		West Midlands			0.12	0.75	0.39
		Chile				0.19	0.46
		Atlanta					0.57
***p* values for comparison of CoV of Mass vs OPv (and CDv)**	CDv		**<0.001**	**0.004**	0.06	**0.04**	0.11
	$OP^{OH}v$		**<0.001**	**0.03**	**0.01**	0.30	0.15
	$OP^{GSH}v$		**<0.001**	**0.005**	**0.05**	**0.002**	**0.03**
	$OP^{C}v$		**<0.001**	**0.01**	0.50	0.15	0.14
	$OP^{DTT}v$		**<0.001**	0.20	0.10	**0.04**	0.10

Supplementary Method 3: Calculation of intrinsic and extrinsic cellular OP

Intrinsic cellular OP ($OP^C m$) was calculated as follows:

OP^C for a $PM_{2.5}$ sample = z%

Blank corrected OP^C = z% - 100% (average of field blank response w.r.t. to Milli-Q) = p%

$OP^C m = p(\%)/m(\mu g)$

m = total mass of $PM_{2.5}$ extract in each well of 96-well plate, which is 6 μg

m is calculated as follows: m = extract concentration in RV x total volume in RV

= 30 μg/mL x 200 μL

= 6 μg

Extrinsic OP^C is calculated as: $OP^C v = OP^C m$ x $PM_{2.5}$ mass concentration (in $\mu g/m^3$)

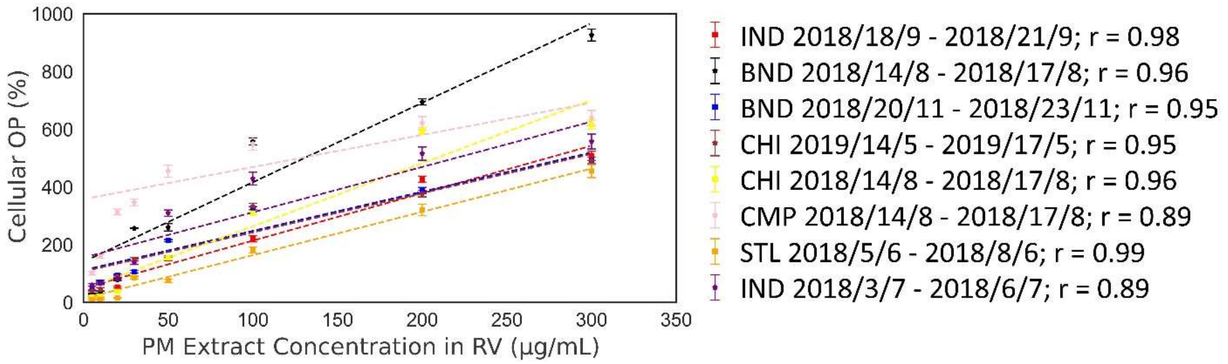

Supplementary Fig. 2: Cellular OP vs $PM_{2.5}$ extract concentration in the reaction vial (RV) for eight randomly selected $PM_{2.5}$ samples. Error bars represent 1 standard deviation. The legend represents the sample IDs of the $PM_{2.5}$ samples used. The sample ID consists of three parts: the site, the start date (yyyy/dd/mm), and the end date of sampling. IND = Indianapolis, BND = Bondville, CHI = Chicago, CMP = Champaign, and STL = St Louis. Figure made using Matplotlib[5].

Supplementary Method 4: Calculation of cytotoxicity

In this study, we calculated intrinsic and extrinsic cytotoxicity (represented as cell death; CD) as follows:

CDm = R/m (%/µg)

R = (100 - CV)

Where,

CV = cell viability

CDm = intrinsic cytotoxicity

R = Cytotoxicity caused by 30 µg/mL of $PM_{2.5}$ extract in RV

m = total mass of $PM_{2.5}$ extract in each well of a 96-well plate, which is 6 µg and is

calculated as follows:

m = extract concentration in RV x total volume in RV

= 30 µg/mL x 200 µL

= 6 µg

Extrinsic cytotoxicity, CDv, is calculated as follows:

CDv = CDm x $PM_{2.5}$ mass concentration (in µg/m^3)

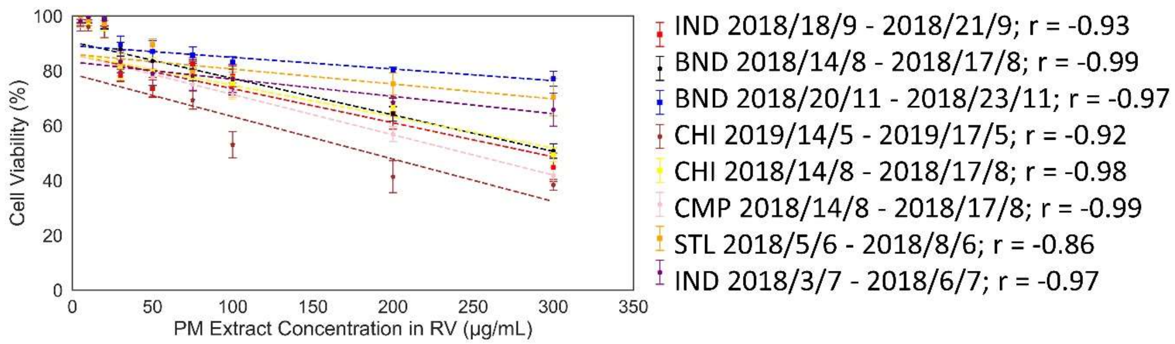

Supplementary Fig. 3: Cell viability vs $PM_{2.5}$ extract concentration in RV for eight randomly selected $PM_{2,5}$ samples. Error bars represent 1 standard deviation. The legend shows the sample IDs of the $PM_{2.5}$ samples, which consist of three parts: the site, the start, and the end date of sampling. IND = Indianapolis, BND = Bondville, CHI = Chicago, CMP = Champaign, and STL = St Louis. Figure made using Matplotlib[5].

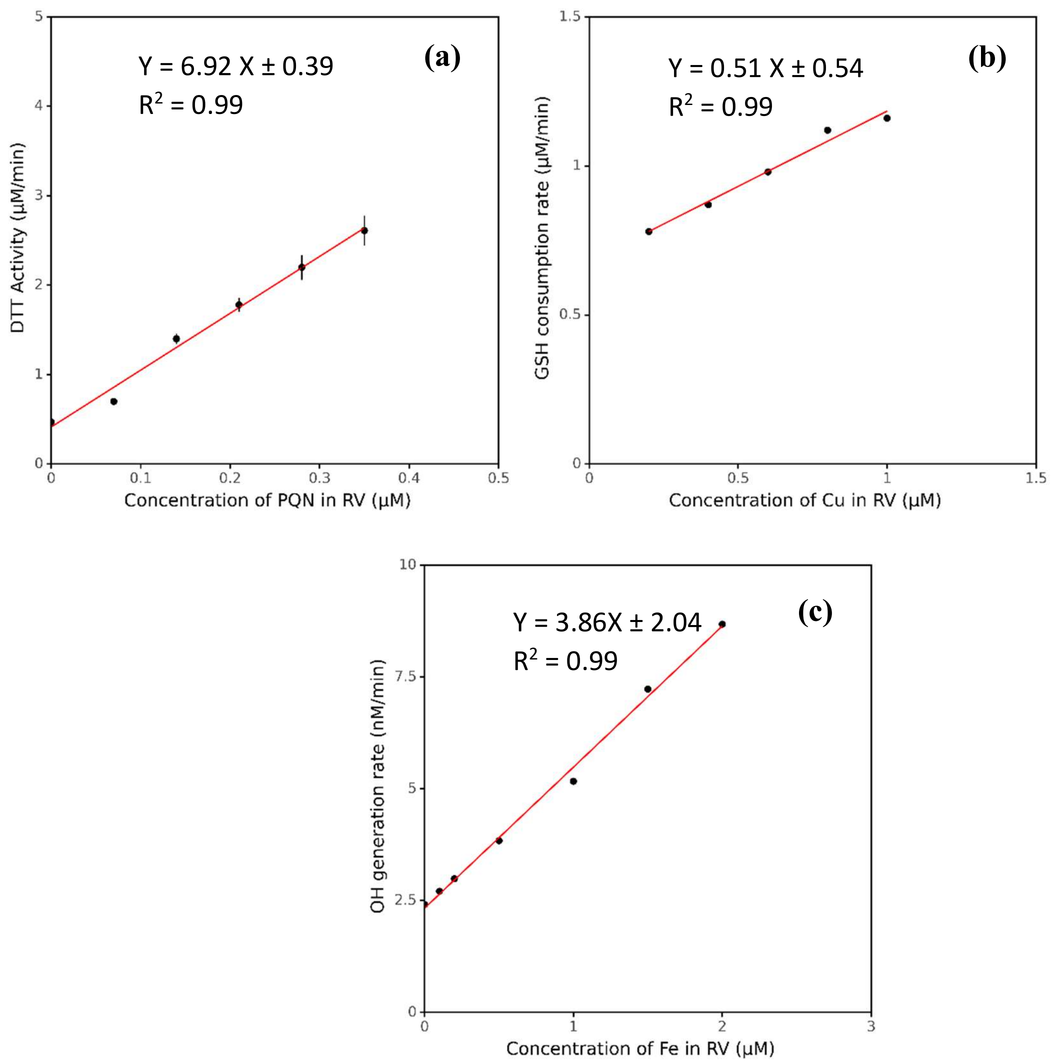

Supplementary Fig. 4: OP as a function of the concentration of positive controls: (a) OP^{DTT} vs. 9,10-Phenanthrenequinone (PQN) concentrations; (b) OP^{GSH} vs. Cu (II) concentrations; (c) OP^{OH} vs. Fe (II) concentrations. Figure made using Matplotlib[5].

Supplementary Method 5: Procedure for normalization of the data ($PM_{2.5}$ mass, OP, and toxicity)

Here, the values for mass, extrinsic OP, and intrinsic OP (and toxicity) were standardized using the min-max scaler technique[4].

In this technique, the data is scaled according to the following formula:

Xstd = (X – Xmin) / (Xmax– Xmin)

Where Xstd = standardized value of a datapoint 'x'

Xmin = minimum value in the entire dataset

Xmax = maximum value in the entire dataset

X= actual value of 'x' in the dataset

(Xmax – Xmin) = range of the values

X- Xmin = distance of X from Xmin

For example, if the min and max observable values in a dataset are 30 and -10, we can normalize any value, like 18, as follows:

y = (x – min) / (max – min)

y = (18 – (-10)) / (30 – (-10))

y = 28 / 40

y = 0.7

We used the MinMaxScaler function in Scikit-Learn, which is a Python library commonly used in statistical and machine learning modelling.

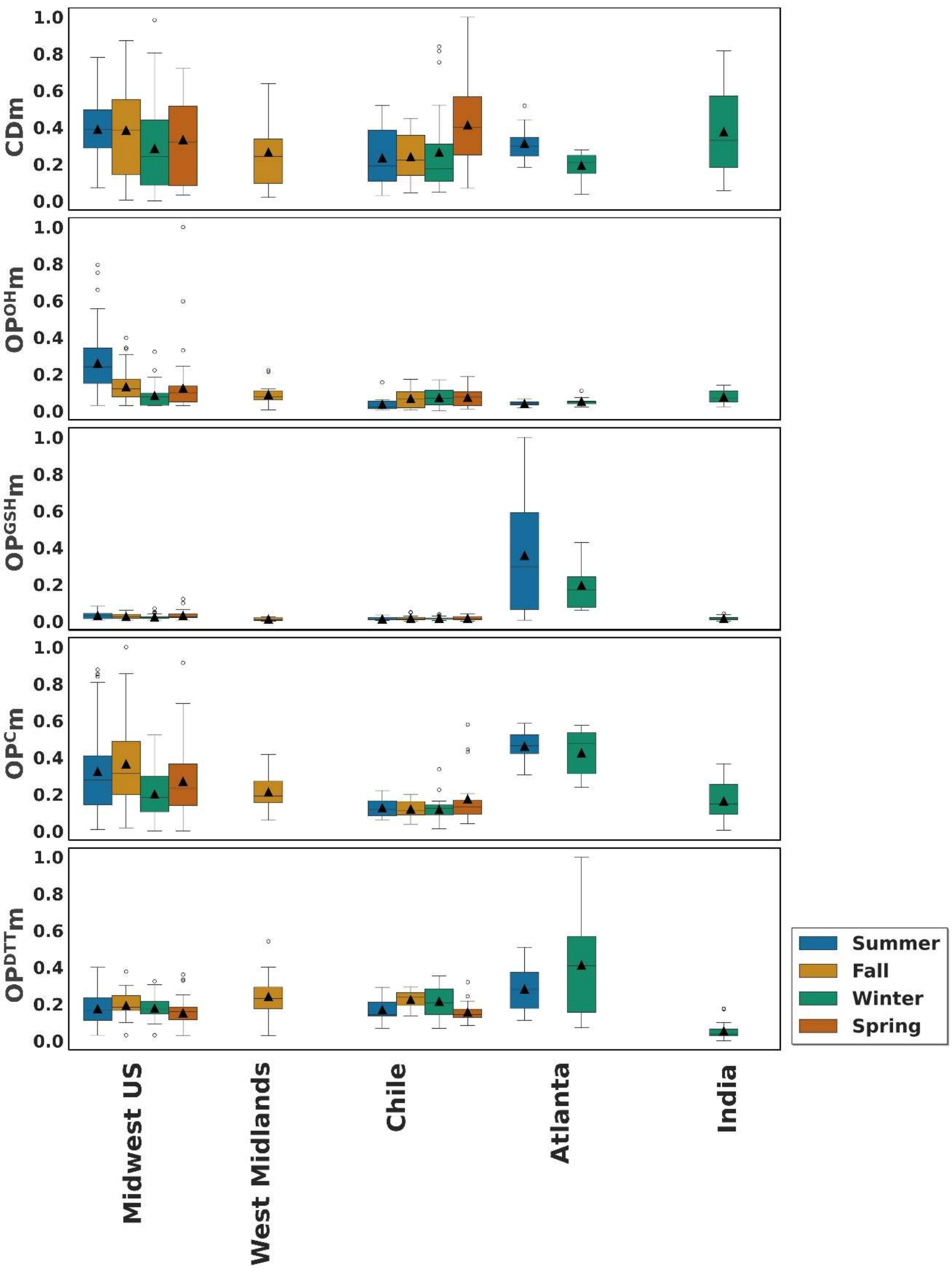

Supplementary Fig. 5: Seasonal distribution of normalized intrinsic cytotoxicity and OP in different geographical regions. The box contains the 25–75th percentile of the measurements, the center line of the box denotes the median, and the whiskers denote 1.5 times the interquartile range. The black triangles represent mean values. Figure made using Seaborn[6].

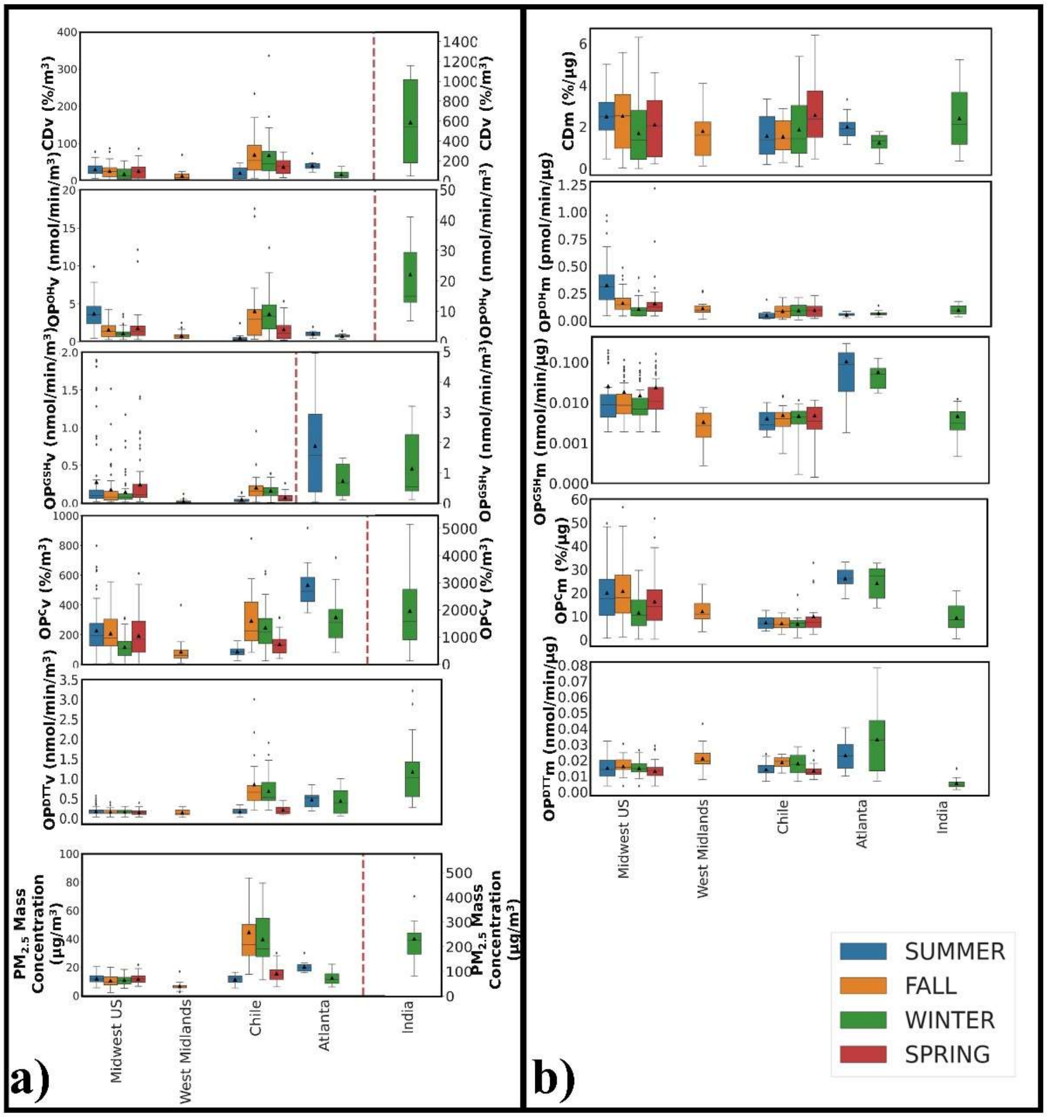

Supplementary Fig. 6: Seasonal distribution of extrinsic (Supplementary Fig. 6a) and intrinsic (Supplementary Fig. 6b) cytotoxicity and OP in different geographical regions. In all panels, the bars on the left-hand side of the red dotted line must be read against the primary Y-axis (on the left-hand side), and the bars on the right-hand side of the red dotted line must be read against the secondary Y-axis (on the right-hand side). The box contains the 25–75th percentile of the measurements, the center line of the box denotes the median, and the whiskers denote 1.5 times the interquartile range. The black triangles represent mean values. Figure made using Seaborn[6].

Supplementary Table 4: Mean, Median, and Standard Deviations (Std. Deviation) of seasonal $PM_{2.5}$ mass concentrations, extrinsic and intrinsic cytotoxicity, and OP at various individual sites.

Site	Season	Statisical Parameter	Extrinsic						Intrinsic				
			Mass	CDv	$OP^{OH}v$	$OP^{GSH}v$	$OP^{C}v$	$OP^{DTT}v$	CDm	$OP^{OH}m$	$OP^{GSH}m$	$OP^{C}m$	$OP^{DTT}m$
Bondville	Summer	Mean	10.37	27.08	4.41	1.32	177.82	0.20	2.65	0.429	0.125	17.61	0.019
		Median	10.08	24.71	3.50	1.39	136.68	0.15	2.18	0.398	0.119	13.96	0.017
		Std. Deviation	2.00	11.59	2.48	0.47	108.34	0.10	1.15	0.219	0.034	10.50	0.007
	Fall	Mean	9.70	15.91	1.29	0.61	119.21	0.15	1.61	0.138	0.060	13.19	0.016
		Median	8.67	9.61	0.97	0.51	105.23	0.14	1.12	0.123	0.062	16.48	0.016
		Std. Deviation	3.52	16.40	1.01	0.38	78.29	0.04	1.32	0.109	0.024	6.97	0.003
	Winter	Mean	8.56	7.60	0.73	0.45	68.38	0.13	1.01	0.092	0.049	8.97	0.015
		Median	7.20	6.43	0.67	0.38	68.75	0.12	0.71	0.097	0.047	8.54	0.015
		Std. Deviation	3.02	6.41	0.29	0.33	38.93	0.05	0.96	0.039	0.021	6.21	0.004
	Spring	Mean	9.19	23.32	1.15	0.71	97.56	0.12	2.44	0.129	0.074	9.91	0.013
		Median	8.70	21.98	0.98	0.72	77.32	0.11	2.93	0.120	0.071	8.06	0.013
		Std. Deviation	2.29	18.77	0.56	0.40	92.27	0.04	1.61	0.069	0.035	7.12	0.003
Champaign	Summer	Mean	11.41	27.96	4.81	0.18	282.48	0.23	2.75	0.466	0.016	29.55	0.020
		Median	11.60	22.76	4.41	0.15	231.00	0.19	2.95	0.447	0.015	26.30	0.019
		Std. Deviation	3.90	14.56	1.83	0.08	184.94	0.12	1.27	0.214	0.005	14.50	0.006
	Fall	Mean	7.47	30.46	1.62	0.11	212.70	0.16	3.90	0.208	0.014	27.54	0.022
		Median	6.29	27.76	1.38	0.10	199.90	0.13	4.46	0.222	0.015	25.69	0.022
		Std. Deviation	4.31	12.08	1.22	0.07	69.38	0.10	1.57	0.084	0.004	11.16	0.005
	Winter	Mean	9.94	16.62	1.30	0.11	154.42	0.19	1.62	0.139	0.011	15.66	0.019
		Median	9.05	15.49	1.11	0.11	126.99	0.18	2.06	0.110	0.011	14.07	0.019
		Std. Deviation	3.02	15.15	0.67	0.04	88.90	0.06	1.23	0.085	0.004	7.38	0.004
	Spring	Mean	11.56	16.29	3.40	0.20	292.60	0.22	1.41	0.168	0.015	25.57	0.018
		Median	10.92	9.31	1.89	0.19	301.85	0.22	1.11	0.161	0.015	21.22	0.017
		Std. Deviation	3.09	14.89	3.80	0.10	91.46	0.07	1.23	0.096	0.002	11.25	0.004
Chicago	Summer	Mean	11.15	25.52	3.98	0.11	204.19	0.19	2.25	0.354	0.010	16.78	0.017
		Median	10.89	22.38	4.03	0.11	160.36	0.15	2.35	0.389	0.009	15.01	0.014
		Std. Deviation	3.18	15.85	1.61	0.03	203.26	0.10	1.06	0.099	0.003	12.58	0.006
	Fall	Mean	10.86	28.42	1.79	0.10	300.47	0.18	2.55	0.175	0.009	28.43	0.017
		Median	10.59	30.72	1.99	0.10	319.75	0.18	2.58	0.127	0.009	26.81	0.017
		Std. Deviation	3.40	24.57	1.01	0.03	158.81	0.05	1.74	0.113	0.003	15.23	0.003
	Winter	Mean	14.58	32.96	0.80	0.08	126.29	0.21	2.46	0.055	0.006	9.36	0.015
		Median	15.39	34.18	0.74	0.09	123.73	0.23	1.88	0.041	0.007	8.65	0.014
		Std. Deviation	3.58	14.00	0.35	0.03	70.51	0.05	1.49	0.025	0.002	6.13	0.003
	Spring	Mean	12.60	35.25	1.66	0.11	253.08	0.15	2.66	0.142	0.009	19.44	0.013
		Median	12.04	26.32	1.47	0.10	202.89	0.14	2.62	0.140	0.010	17.41	0.014
		Std. Deviation	4.16	24.49	0.83	0.06	176.38	0.06	1.21	0.076	0.003	10.81	0.004
Indianapolis	Summer	Mean	11.88	24.64	2.24	0.06	214.09	0.14	2.02	0.184	0.005	17.44	0.012
		Median	11.60	24.78	1.38	0.04	188.02	0.12	2.22	0.142	0.004	18.34	0.011
		Std. Deviation	3.48	13.73	1.64	0.07	148.73	0.11	0.77	0.125	0.004	12.50	0.008
	Fall	Mean	11.45	27.54	1.50	0.06	201.93	0.16	2.70	0.125	0.005	19.91	0.014
		Median	10.36	25.09	1.45	0.04	185.14	0.15	2.88	0.133	0.006	17.01	0.014
		Std. Deviation	4.21	18.57	0.86	0.04	128.93	0.06	1.43	0.061	0.003	11.51	0.003
	Winter	Mean	11.03	23.55	1.19	0.05	101.90	0.14	2.46	0.118	0.005	10.43	0.012
		Median	10.93	32.19	1.18	0.05	96.43	0.15	2.59	0.118	0.005	8.65	0.013
		Std. Deviation	2.71	18.73	0.59	0.02	51.31	0.06	2.16	0.053	0.002	7.41	0.006
	Spring	Mean	12.21	26.68	1.26	0.07	149.92	0.12	2.12	0.103	0.006	12.42	0.011
		Median	12.16	28.65	1.32	0.07	119.91	0.11	2.42	0.111	0.006	10.24	0.010
		Std. Deviation	2.11	18.33	0.93	0.03	103.94	0.06	1.31	0.065	0.002	10.48	0.004
St Louis	Summer	Mean	14.74	41.98	3.31	0.07	246.89	0.14	2.57	0.247	0.005	15.66	0.010
		Median	15.08	36.39	3.24	0.06	222.59	0.13	2.55	0.253	0.004	16.53	0.008
		Std. Deviation	3.40	19.17	1.45	0.05	142.01	0.10	0.75	0.114	0.003	5.95	0.007
	Fall	Mean	13.07	24.82	1.77	0.05	207.22	0.16	2.17	0.103	0.003	18.37	0.012
		Median	12.43	19.86	1.23	0.04	190.35	0.16	2.01	0.041	0.002	20.30	0.013
		Std. Deviation	3.71	19.51	1.51	0.03	106.72	0.06	1.51	0.096	0.002	7.69	0.004
	Winter	Mean	11.77	14.66	1.17	0.06	129.15	0.15	1.25	0.092	0.005	11.05	0.014
		Median	11.45	8.71	1.02	0.06	115.60	0.15	0.76	0.084	0.005	11.37	0.014
		Std. Deviation	2.80	14.13	0.86	0.02	86.07	0.06	1.13	0.067	0.002	8.37	0.003
	Spring	Mean	13.79	22.91	1.22	0.08	182.96	0.12	1.94	0.079	0.006	13.00	0.009
		Median	12.70	19.35	1.26	0.09	142.41	0.15	1.73	0.076	0.006	8.23	0.010
		Std. Deviation	4.04	19.71	0.54	0.04	156.30	0.07	1.24	0.042	0.003	11.24	0.005

Supplementary Table 4 (Contd.):

Site	Season	Statisical Parameter	Extrinsic						Intrinsic				
			Mass	CDv	$OP^{OH}v$	$OP^{GSH}v$	$OP^{C}v$	$OP^{DTT}v$	CDm	$OP^{OH}m$	$OP^{GSH}m$	$OP^{C}m$	$OP^{DTT}m$
**BROS**	**Fall**	Mean	7.14	9.48	1.01	0.02	81.71	0.16	1.35	0.143	0.003	11.28	0.022
		Median	6.90	7.47	0.86	0.02	81.65	0.15	1.22	0.116	0.003	11.01	0.021
		Std. Deviation	1.21	7.54	0.52	0.01	29.78	0.09	1.14	0.081	0.002	3.39	0.012
**EROS**		Mean	6.26	14.82	0.65	0.03	89.76	0.13	1.93	0.091	0.004	11.83	0.021
		Median	5.47	9.31	0.54	0.02	54.71	0.12	1.96	0.091	0.003	10.62	0.019
		Std. Deviation	3.64	17.83	0.64	0.03	101.00	0.08	1.32	0.048	0.003	5.90	0.006
**Santiago**	**Summer**	Mean	12.73	24.02	0.30	0.06	85.87	0.21	1.80	0.023	0.004	6.61	0.016
		Median	12.82	24.14	0.23	0.03	77.90	0.21	2.04	0.020	0.003	6.16	0.015
		Std. Deviation	2.52	15.65	0.16	0.05	39.87	0.07	1.09	0.010	0.003	2.59	0.005
	**Fall**	Mean	39.87	69.64	3.65	0.26	293.73	0.69	1.73	0.094	0.006	7.29	0.017
		Median	36.39	66.39	3.68	0.17	251.14	0.65	1.65	0.110	0.005	7.18	0.017
		Std. Deviation	13.19	38.63	1.70	0.28	150.54	0.27	0.84	0.042	0.005	2.70	0.003
	**Winter**	Mean	40.85	56.76	3.05	0.17	238.56	0.67	1.44	0.074	0.005	5.82	0.017
		Median	33.65	45.23	3.23	0.15	219.04	0.48	1.13	0.060	0.004	6.56	0.014
		Std. Deviation	18.69	42.65	2.22	0.09	161.81	0.47	1.03	0.049	0.003	2.69	0.007
	**Spring**	Mean	19.38	41.44	1.79	0.11	123.25	0.29	1.93	0.088	0.006	5.91	0.015
		Median	17.36	37.61	1.35	0.12	112.05	0.29	1.62	0.092	0.005	6.24	0.015
		Std. Deviation	5.88	23.60	1.66	0.09	65.15	0.11	0.95	0.060	0.005	2.08	0.005
**Chillan**	**Summer**	Mean	8.69	7.71	0.92	0.03	95.96	0.09	0.75	0.095	0.003	9.46	0.010
		Median	8.33	5.66	0.59	0.02	104.37	0.12	0.68	0.072	0.003	9.72	0.010
		Std. Deviation	3.07	6.56	0.86	0.01	16.22	0.05	0.60	0.055	0.002	3.22	0.003
	**Fall**	Mean	49.43	69.37	4.37	0.15	295.49	1.02	1.34	0.079	0.004	6.53	0.020
		Median	32.82	34.97	0.96	0.14	179.58	0.62	1.20	0.045	0.003	6.43	0.021
		Std. Deviation	40.93	76.46	6.76	0.10	247.36	0.93	0.93	0.078	0.002	2.47	0.004
	**Winter**	Mean	37.16	90.07	4.78	0.15	269.45	0.71	2.72	0.128	0.005	8.47	0.021
		Median	32.16	45.10	4.89	0.13	209.06	0.69	2.88	0.148	0.006	7.28	0.022
		Std. Deviation	21.57	100.01	3.77	0.12	149.82	0.33	2.02	0.064	0.002	4.49	0.006
	**Spring**	Mean	11.91	33.08	1.43	0.04	151.48	0.14	2.47	0.106	0.003	12.70	0.012
		Median	11.28	35.56	0.94	0.04	132.11	0.11	1.89	0.104	0.003	9.72	0.012
		Std. Deviation	3.99	20.49	1.43	0.02	99.66	0.05	1.95	0.067	0.002	8.99	0.002
**Atlanta**	**Summer**	Mean	20.30	40.26	1.05	1.89	534.38	0.47	2.00	0.053	0.104	26.15	0.023
		Median	19.50	38.06	0.98	1.58	493.43	0.52	1.91	0.053	0.086	26.25	0.023
		Std. Deviation	4.11	14.29	0.43	1.76	167.45	0.22	0.66	0.019	0.101	4.72	0.010
	**Winter**	Mean	12.55	16.44	0.78	0.74	318.20	0.44	1.23	0.067	0.057	24.15	0.033
		Median	11.25	12.92	0.80	0.67	282.79	0.39	1.33	0.064	0.050	27.05	0.033
		Std. Deviation	5.28	11.50	0.30	0.55	196.95	0.34	0.49	0.030	0.040	7.44	0.023
**Hisar**	**Winter**	Mean	244.30	508.90	24.55	1.35	2089.53	0.74	1.99	0.113	0.005	9.33	0.004
		Median	201.93	352.78	29.15	0.49	2091.62	0.71	2.07	0.134	0.004	8.93	0.004
		Std. Deviation	185.30	453.87	14.24	1.41	1357.63	0.35	1.27	0.054	0.004	4.69	0.002
**Faridabad**		Mean	170.99	154.97	11.63	0.49	971.34	0.50	0.99	0.078	0.003	7.61	0.004
		Median	161.70	169.48	13.38	0.48	909.08	0.54	0.89	0.085	0.003	5.65	0.003
		Std. Deviation	80.42	49.98	3.61	0.31	599.40	0.08	0.35	0.033	0.001	5.92	0.002
**Ahmedabad**		Mean	221.00	1047.95	19.98	1.62	4083.24	2.78	4.77	0.088	0.008	18.40	0.013
		Median	221.00	1013.60	22.75	1.96	3683.98	2.88	4.58	0.092	0.008	18.99	0.015
		Std. Deviation	27.00	60.44	11.11	0.95	934.34	0.50	0.39	0.047	0.005	2.71	0.003
**Patiala**		Mean	288.60	821.92	31.48	1.30	1570.11	1.32	2.71	0.113	0.003	5.81	0.005
		Median	258.00	799.62	24.40	0.54	1414.06	1.34	2.64	0.114	0.002	5.77	0.004
		Std. Deviation	70.16	305.49	20.91	1.31	694.45	0.20	0.83	0.048	0.002	3.39	0.001

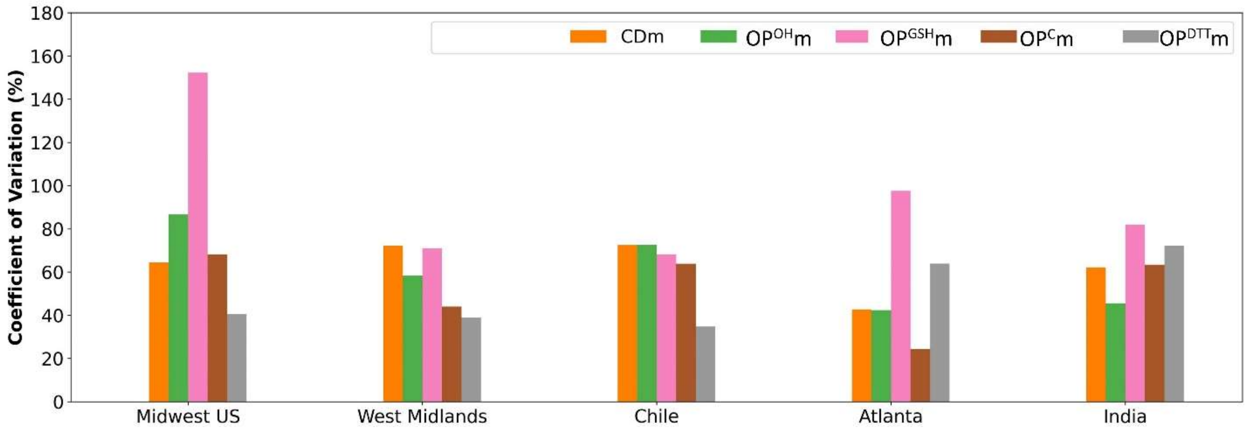

Supplementary Fig. 7: Coefficient of Variation (CoV) for various intrinsic $PM_{2.5}$ endpoints, i.e., acellular and cellular OP, and CD at different geographical regions. Figure made using Matplotlib[5].

Supplementary Table 5: Pearson's *r* for the correlation of ambient concentrations of different $PM_{2.5}$ chemical species with extrinsic OP and cytotoxicity

Midwest US

	CDv	$OP^{OH}v$	$OP^{GSH}v$	$OP^{C}v$	$OP^{DTT}v$
Al	0.27	0.48	0.28	0.27	0.47
As	0.19	0.26	-0.05	0.25	0.12
Ba	0.22	0.18	-0.03	0.15	0.24
Cd	0.24	0.33	0.11	0.30	0.20
Co	0.25	0.23	-0.05	0.23	0.11
Cr	0.21	0.33	0.33	0.23	0.23
Cu	0.13	0.55	0.03	0.36	0.50
Fe	0.28	0.50	0.17	0.36	0.20
Ga	0.23	0.18	-0.04	0.15	0.24
K	0.18	0.17	0.02	0.12	0.24
Li	0.23	0.19	-0.16	0.35	0.21
Mn	0.31	0.29	-0.05	0.41	0.18
Ni	0.07	0.02	0.02	0.06	-0.03
Pb	0.16	0.30	0.25	0.17	-0.01
Rb	0.25	0.26	-0.01	0.34	0.38
Sr	0.18	0.16	0.00	0.12	0.22
V	0.06	0.06	-0.09	0.08	0.08
Zn	0.21	0.17	0.16	0.25	0.25
WSOC	0.13	0.04	-0.18	0.06	-0.04

West Midlands

	CDv	$OP^{OH}v$	$OP^{GSH}v$	$OP^{C}v$	$OP^{DTT}v$
Al	0.27	0.65	0.28	0.25	0.19
As	0.46	0.29	0.28	0.51	0.35
Ba	0.29	0.27	0.27	0.45	0.47
Cd	0.55	0.50	0.52	0.66	0.64
Co	0.33	0.77	0.45	0.48	0.55
Cr	0.24	0.72	0.40	0.42	0.54
Cu	0.81	0.65	0.78	0.91	0.61
Fe	0.40	0.78	0.51	0.57	0.61
Ga	0.31	0.31	0.29	0.46	0.48
K	0.38	0.31	0.30	0.55	0.44
Li	0.66	0.65	0.69	0.76	0.70
Mn	0.70	0.74	0.81	0.82	0.71
Ni	0.48	0.69	0.52	0.56	0.57
Pb	0.36	0.21	0.14	0.37	0.04
Rb	0.66	0.77	0.67	0.82	0.62
Sr	0.20	0.08	0.06	0.32	0.22
V	0.64	0.72	0.80	0.78	0.73
Zn	0.66	0.49	0.52	0.72	0.48
WSOC	-0.16	-0.21	-0.20	-0.14	-0.21

Chile

	CDv	$OP^{OH}v$	$OP^{GSH}v$	$OP^{C}v$	$OP^{DTT}v$
Al	0.21	0.54	0.34	0.56	0.54
As	0.44	0.65	0.44	0.61	0.80
Ba	0.28	0.40	0.42	0.36	0.53
Cd	0.25	0.31	0.44	0.65	0.75
Co	0.14	0.25	0.21	0.32	0.39
Cr	0.13	0.23	0.24	0.29	0.34
Cu	0.37	0.43	0.43	0.56	0.75
Fe	0.14	0.24	0.21	0.29	0.35
Ga	0.26	0.38	0.42	0.35	0.52
K	0.36	0.62	0.41	0.54	0.71
Li	0.34	0.48	0.24	0.56	0.69
Mn	0.31	0.44	0.37	0.59	0.70
Ni	0.15	0.25	0.24	0.32	0.37
Pb	0.39	0.47	0.49	0.59	0.73
Rb	0.42	0.72	0.28	0.59	0.74
Sr	0.35	0.52	0.41	0.50	0.70
V	0.18	0.33	0.34	0.33	0.37
Zn	0.31	0.47	0.50	0.48	0.63
WSOC	0.51	0.35	0.17	0.33	0.38

Atlanta

	CDv	$OP^{OH}v$	$OP^{GSH}v$	$OP^{C}v$	$OP^{DTT}v$
Al	0.26	0.10	0.33	0.13	0.07
As	-0.10	-0.10	-0.25	-0.12	-0.03
Ba	-0.01	0.41	0.19	-0.21	0.21
Cd	-0.37	-0.09	-0.25	-0.44	-0.21
Co	0.17	0.24	0.26	0.17	0.06
Cr	0.19	0.23	0.17	0.27	0.07
Cu	0.47	0.63	-0.18	0.48	0.44
Fe	0.65	0.33	0.21	0.75	0.66
Ga	-0.03	0.35	0.23	-0.23	0.16
K	0.44	0.18	0.34	0.42	0.36
Li	0.03	0.18	0.15	-0.11	0.13
Mn	0.19	0.15	0.21	0.06	0.05
Ni	0.07	0.22	0.37	0.11	-0.06
Pb	0.08	0.24	-0.13	0.13	0.40
Rb	-0.29	0.11	0.11	-0.38	0.08
Sr	0.06	0.08	0.44	0.04	0.22
V	0.22	0.12	0.27	0.20	-0.03
Zn	0.55	0.37	0.28	0.30	0.22
WSOC	0.55	0.44	-0.08	0.33	0.09

India

	CDv	$OP^{OH}v$	$OP^{GSH}v$	$OP^{C}v$	$OP^{DTT}v$
Al	-0.18	-0.07	0.19	-0.24	-0.17
As	0.29	0.28	0.64	0.13	0.42
Ba	0.28	0.27	0.57	0.01	0.42
Cd	-0.08	-0.34	-0.27	0.18	0.11
Co	0.46	-0.14	0.27	0.66	0.84
Cr	0.21	0.00	0.10	0.05	0.10
Cu	-0.21	0.19	0.28	0.23	-0.13
Fe	0.24	-0.14	0.03	0.34	0.59
Ga	0.29	0.27	0.57	0.01	0.42
K	0.23	0.38	0.48	-0.11	0.14
Li	0.39	0.04	0.38	0.70	0.46
Mn	0.44	-0.07	0.24	0.73	0.65
Ni	-0.04	-0.02	-0.17	-0.11	0.003
Pb	-0.05	-0.02	-0.17	-0.12	-0.02
Rb	0.53	-0.06	0.51	0.41	0.80
Sr	0.37	0.20	0.27	0.31	0.69
V	0.50	0.16	-0.01	0.29	0.36
Zn	0.27	0.05	-0.09	0.26	0.25
WSOC	0.42	0.72	0.51	0.10	0.01

-0.4 -0.2 0 0.1 0.2 0.3 0.4 0.5 0.6 0.7 0.8 0.9 1

	Site	Mass	CDv	$OP^{OH}v$	$OP^{GSH}v$	$OP^{C}v$	$OP^{DTT}v$	Al	As	Ba	Cd	Co	Cr	Cu	Fe	Ga	K	Li	Mn	Ni	Pb	Rb	Sr	V	Zn	WSOC
CoV based on Mean (%) = Standard Deviation×100/Mean	Bondville	29	85	105	65	79	45	146	59	87	79	119	114	107	94	75	56	53	90	224	125	49	71	62	104	58
	Champaign	38	68	89	56	56	47	101	57	52	54	154	38	94	73	46	56	49	61	86	65	52	83	69	107	61
	Chicago	31	68	71	42	76	42	110	49	116	67	79	46	70	58	103	178	73	79	104	93	71	246	67	51	47
	Indianapolis	27	66	74	79	72	56	162	59	325	44	77	84	193	56	295	397	34	53	552	68	71	514	69	41	45
	St Louis	26	80	74	60	70	51	121	50	236	41	51	65	106	73	207	308	43	36	70	135	53	297	128	51	42
	BROS	17	79	52	72	36	58	51	74	53	39	47	52	33	37	50	60	24	23	50	55	35	91	33	43	18
	EROS	58	120	98	122	113	57	66	81	59	56	102	120	33	97	60	57	61	69	117	77	68	55	62	62	75
	Santiago	59	65	91	100	75	75	37	75	29	75	141	123	82	141	28	27	71	93	139	78	33	24	75	33	35
	Chillan	100	77	139	101	79	118	142	141	74	198	151	255	136	160	75	112	107	144	150	130	149	93	126	82	45
	Atlanta	37	62	42	106	49	62	63	87	36	192	49	50	76	29	36	36	53	51	66	49	49	106	44	53	46
	India	49	71	68	95	69	74	122	108	194	52	164	36	98	54	194	98	46	108	113	113	116	172	91	82	56

	Site	Mass	CDv	$OP^{OH}v$	$OP^{GSH}v$	$OP^{C}v$	$OP^{DTT}v$	Al	As	Ba	Cd	Co	Cr	Cu	Fe	Ga	K	Li	Mn	Ni	Pb	Rb	Sr	V	Zn	WSOC
Robust CoV (RCV_Q) based on median (m) and Interquartile Range (IQR) (%) = 0.75 × IQR×100/m	Bondville	36	145	114.1	65.5	69.3	42.2	95	68	41	74	57	62	81	76	44	45	52	79	98	67	65	58	63	42	58
	Champaign	44	81	139.9	47.2	59.7	41	94	64	32	43	67	44	132	89	35	46	47	59	57	65	56	60	68	50	66
	Chicago	32	67	79	39.7	81.8	44.5	66	45	38	53	84	38	102	68	42	41	71	61	120	94	92	54	92	56	44
	Indianapolis	23	67	74	58.5	59.8	47	68	55	44	45	60	35	56	65	41	33	31	50	47	86	34	47	63	45	42
	St Louis	32	90	108.4	61.4	57.7	51.5	88	37	53	45	59	25	47	81	52	37	56	36	103	96	35	58	135	51	66
	BROS	19	106	62.5	62.8	33.6	82.8	40	77	100	52	57	66	52	42	92	118	25	19	65	58	42	250	31	46	21
	EROS	29	92	74	100	63	54	98	128	57	46	84	95	69	88	58	55	47	67	87	85	59	55	38	50	54
	Santiago	59	86	128	64	86	62	47	83	30	71	114	163	77	125	30	22	38	77	115	69	33	26	70	27	30
	Chillan	103	107	249	160	74	187	157	243	96	127	140	137	128	269	100	163	153	123	168	144	176	132	198	109	47
	Atlanta	41	70	41	204	51	69	29	81	28	139	42	44	98	35	28	41	44	49	43	60	49	45	38	59	21
	India	33	118	86	272	96	67	150	67	522	83	49	25	63	36	538	60	48	50	68	69	200	402	114	77	51

	Site	Mass	CDv	$OP^{OH}v$	$OP^{GSH}v$	$OP^{C}v$	$OP^{DTT}v$	Al	As	Ba	Cd	Co	Cr	Cu	Fe	Ga	K	Li	Mn	Ni	Pb	Rb	Sr	V	Zn	WSOC
Robust CoV (RCV_M) based on Median Absolute Deviation (MAD) and m (%) = 1.4826 × MAD ×100/m	Bondville	33	101	71	70	68	37	90	64	41	63	57	36	68	70	40	40	49	70	63	65	54	50	64	38	54
	Champaign	39	77	82	37	60	40	84	53	32	44	60	32	88	78	35	38	42	58	56	66	43	60	63	47	49
	Chicago	34	67	76	39	81	39	64	41	39	48	68	31	68	69	34	36	59	57	72	85	66	40	72	45	46
	Indianapolis	22	65	73	55	57	45	65	53	40	48	61	37	54	64	40	32	30	43	46	75	33	43	60	37	42
	St Louis	31	88	94	62	57	41	77	39	52	43	56	26	40	87	48	35	57	35	73	62	34	56	81	45	62
	BROS	16	106	63	63	34	83	41	62	54	47	52	65	47	38	42	61	21	15	61	36	35	77	16	49	13
	EROS	26	92	74	100	63	54	46	88	52	56	77	92	62	85	52	50	53	66	81	83	56	54	36	44	64
	Santiago	66	76	122	69	78	65	53	83	28	78	107	132	77	120	28	16	40	73	113	66	33	27	69	27	28
	Chillan	73	104	103	92	52	97	141	145	88	127	127	137	123	137	94	143	133	123	130	123	143	132	141	104	33
	Atlanta	39	65	35	120	47	75	28	66	28	63	48	35	51	36	25	42	37	49	47	56	46	33	32	57	20
	India	26	103	78	98	70	69	122	58	88	55	41	24	44	35	90	55	46	31	75	65	100	101	114	64	53

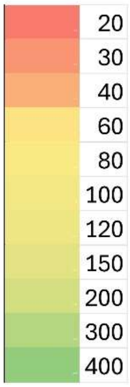

Supplementary Fig. 8: Heatmap showing three different metrics of variability [CoV, RCV_Q, and RCV_M (%)] for various extrinsic $PM_{2.5}$ endpoints, i.e., mass, volume normalized acellular and cellular OP, and CD, and ambient concentrations of various $PM_{2.5}$ chemical species ($\mu g/m^3$) at individual sampling sites. RCV_Q represents robust coefficient of variation based on the interquartile range and RCV_M represents robust coefficient of variation based on the median. For India, we have clubbed all the sites together because the number of $PM_{2.5}$ samples from each site is much lower (n < 5) as compared to all other sites (n > 10). (Ahmedabad, n =3; Patiala, n = 5; Hisar, n = 5; Faridabad, n = 5).

Supplementary Discussion 1: Correlation among different OP and toxicity endpoints

We conducted a correlation analysis among all OP and cytotoxicity endpoints. The heatmap with the correlation coefficients (Pearson's *r*) for different regions is shown in Supplementary Fig. 9. The figure shows that there was no appreciable correlation among most of the intrinsic endpoints, except for the correlation between $OP^{C}m$ and CDm ($r > 0.5$). The correlations among extrinsic endpoints were better, although no consistent pattern was observed across different regions. For example, CDv showed a strong correlation with $OP^{C}v$ ($r > 0.6$) and a moderate correlation with almost all acellular OP ($0.4 < r < 0.6$) in most of the regions, except Midwest US. On the other hand, $OP^{GSH}v$ and $OP^{OH}v$ showed a weak correlation ($r < 0.4$) with other endpoints at most of the sites except West Midlands ($r > 0.4$). A more detailed discussion about these correlations and their mechanistic explanation is beyond the scope of the current paper.

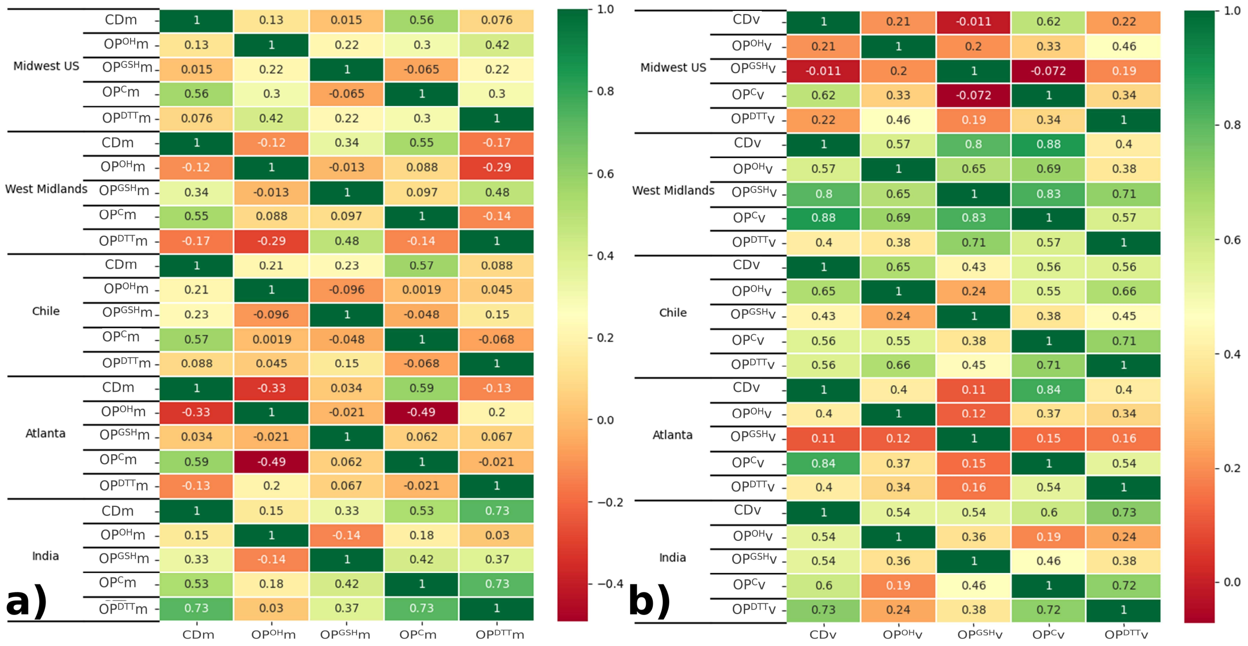

Supplementary Fig. 9: Heatmaps showing correlations (Pearson's *r*) among different OP and cytotoxicity endpoints for different regions. Panel a) shows correlations among intrinsic endpoints and b) shows correlations among extrinsic endpoints. Figure made using Matplotlib[5].

Supplementary Method 6: Details regarding the logistic curves to explain the relationship between extrinsic OP (and cytotoxicity) vs. $PM_{2.5}$ mass concentrations.

We used the optimization package from an open-source Python library called SciPy which is commonly used for solving scientific and technical computing problems such as optimization, integration, linear algebra, and constructing special functions. A logistic function is defined as:

$$Y = \frac{L}{1+e^{(-k(x-x0))}} + b$$

Where,

x0 is the value of the midpoint of the logistic function.

k = the logistic growth rate of the function

L = the maximum value of the Y in each iteration

b = bias in the function

Note, x is the dataset containing $PM_{2.5}$ mass concentrations and Y is the predicted value of OP or cytotoxicity.

The initial guesses for the different parameters were as follows:

L = maximum value in y dataset [i.e., maximum value in the OPv (or CDv) dataset]

k = 0.1 for OP^{GSH}v and 1 for all others

x0 = median of x dataset (i.e., median of $PM_{2.5}$ mass concentrations)

b = minimum value in y dataset

number of iterations = 4000

Supplementary Method 7: Water-soluble organic carbon (WSOC) and elemental analysis

For WSOC analysis, the water-soluble $PM_{2.5}$ extracts were first diluted by DI to an appropriate concentration (50 $\mu g\ mL^{-1}$) and transferred to a 30 mL glass bottle (rinsed with DI and baked at 550 ^oC for 24 h to remove organic residues, if any). Then a mixture of 25 mL of DI and 25 µL of HCl was added to the glass bottle to acidify the sample (pH < 2). Before the analysis of each batch of samples (n ≈ 20), a calibration curve was first prepared using TOC standards of different concentrations (0, 1, 1.25, 1.67, 2.5 and 5 ppm prepared using 1000 mg L^{-1} stock TOC standard obtained from Aqua Solutions, Inc., Deer Park, Texas). The calibration curve was then used to calculate the WSOC concentration in each sample. For ICP-MS, the water-soluble $PM_{2.5}$ extract samples were acidified with 0.5% nitric acid prior to analysis. Before the analysis of each batch of samples (n ≈ 30), a calibration curve was prepared by diluting a multi-element calibration standard (concentration of each elemental species = 10 ppm; obtained from Perkin Elmer) to various concentrations (0, 5, 10, 20, 40, 80 ppb).

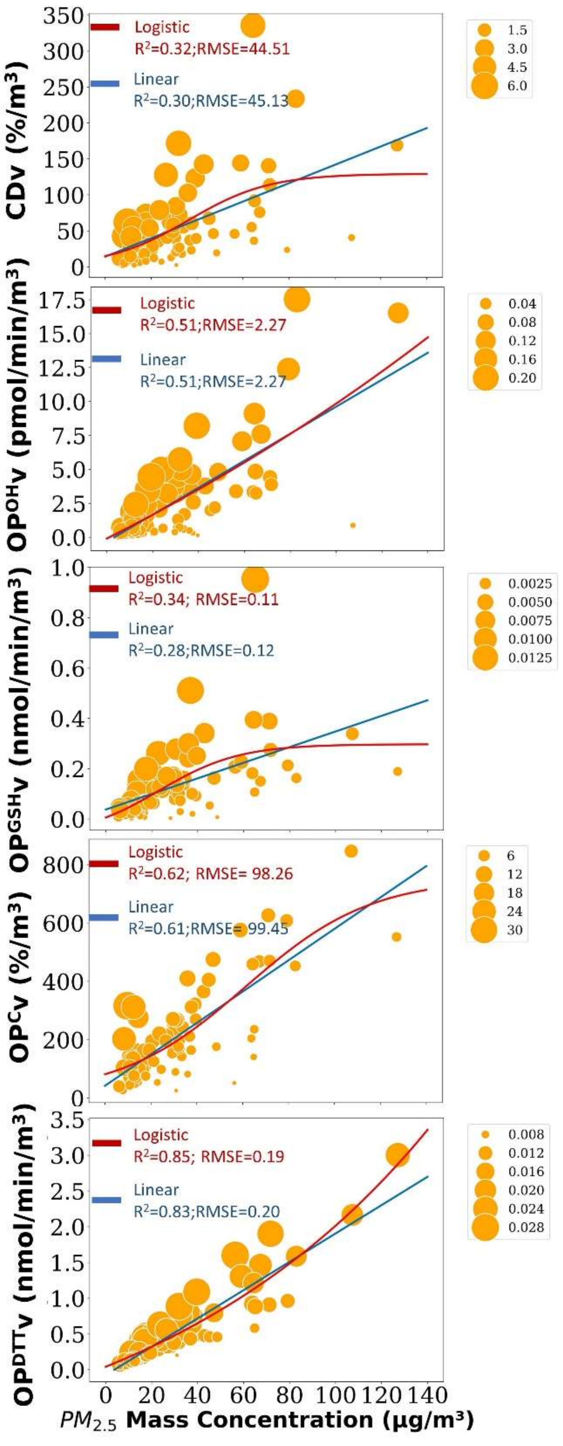

Supplementary Fig. 10: Relationship between extrinsic OP or cytotoxicity (Y-Axis) vs. $PM_{2.5}$ mass concentrations (X-Axis) for Chile. The intrinsic OP and cytotoxicity are represented by the size of the bubble. For each endpoint, the blue line represents the linear curve, and the red line represents the logistic curve. RMSE represents Root Mean Squared Error. Figure made using Seaborn[6].

Supplementary References

(1) Klein, C. B.; Su, L.; Bowser, D.; Leszcynska, J. Chromate-induced epimutations in mammalian cells. *Environ. Health Perspect.* **2002**, *110* (SUPPL. 5), 739–743.

(2) Reiniers, M. J.; Van Golen, R. F.; Bonnet, S.; Broekgaarden, M.; Van Gulik, T. M.; Egmond, M. R.; Heger, M. Preparation and practical Applications of 2′,7′-Dichlorodihydrofluorescein in redox assays. *Anal. Chem.* **2017**, *89* (7), 3853–3857.

(3) Yu, H.; Puthussery, J. V.; Verma, V. A semi-automated multi-endpoint reactive oxygen species activity analyzer (SAMERA) for measuring the oxidative potential of ambient PM2.5 aqueous extracts. *Aerosol Sci. Technol.* **2019**, *54* (3), 304–320.

(4) Jain, A.; Nandakumar, K.; Ross, A. Score normalization in multimodal biometric systems. *Pattern Recognit.* **2005**, *38* (12), 2270–2285.

(5) Hunter, J.D. Matplotlib: A 2D graphics environment. *Comput. Sci. Eng.* **2007**, *9* (3), 90-95.

(6) Waskom, M. L. Seaborn: Statistical data visualization. *J. Open Source Softw.* **2021**, *6* (60), 3021.

(7) Plotly Technologies Inc. Collaborative data science. *Plotly Technologies Inc.* **2015.** https://plot.ly
